# Patch utilization and flower visitations by wild bees in a honey bee‐dominated, grassland landscape

**DOI:** 10.1002/ece3.8174

**Published:** 2021-10-10

**Authors:** Clint R. V. Otto, Larissa L. Bailey, Autumn H. Smart

**Affiliations:** ^1^ U.S. Geological Survey Northern Prairie Wildlife Research Center Jamestown North Dakota USA; ^2^ Department of Fish, Wildlife and Conservation Biology Colorado State University Fort Collins Colorado USA; ^3^ Department of Entomology University of Nebraska Lincoln Nebraska USA

**Keywords:** dietary niche, forage, habitat, honey bee, managed bee, native bee, occupancy

## Abstract

Understanding habitat needs and patch utilization of wild and managed bees has been identified as a national research priority in the United States. We used occupancy models to investigate patterns of bee use across 1030 transects spanning a gradient of floral resource abundance and richness and distance from apiaries in the Prairie Pothole Region (PPR) of the United States. Estimates of transect use by honey bees were nearly 1.0 during our 3.5‐month sampling period, suggesting honey bees were nearly ubiquitous across transects. Wild bees more frequently used transects with higher flower richness and more abundant flowers; however, the effect size of the native flower abundance covariate (${\hat{\beta }}_{\mathrm{native}}$ = 3.90 ± 0.65 [1SE]) was four times greater than the non‐native flower covariate (${\hat{\beta }}_{non‐native}$ = 0.99 ± 0.17). We found some evidence that wild bee use was lower at transects near commercial apiaries, but the effect size was imprecise (${\hat{\beta }}_{\mathrm{distance}}$ = 1.4 ± 0.81). Honey bees were more frequently detected during sampling events with more non‐native flowers and higher species richness but showed an uncertain relationship with native flower abundance. Of the 4039 honey bee and flower interactions, 85% occurred on non‐native flowers, while only 43% of the 738 wild bee observations occurred on non‐native flowers. Our study suggests wild bees and honey bees routinely use the same resource patches in the PPR but often visit different flowering plants. The greatest potential for resource overlap between honey bees and wild bees appears to be for non‐native flowers in the PPR. Our results are valuable to natural resource managers tasked with supporting habitat for managed and wild pollinators in agroecosystems.

## Graphical abstract

We investigated use of grassland patches by wild bees and honey bees. Our study showed that while honey bees and wild bees commonly co‐occurred at the same resource patches, they often visited different species of flowers within patches.

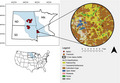

## 1 INTRODUCTION

Pollinator declines have generated significant public interest in conserving bees and their habitats (Goulson et al., 2015; Hall & Martins, 2020; Spivak et al., 2017). In the United States, multiple government and nongovernment initiatives have been developed to enhance bee habitat on public and private lands (Pollinator Health Task Force, 2015). The U.S. Department of Agriculture released a research action plan in 2021 highlighting the need for additional habitat and forage research of managed and wild bees to ensure habitat needs of both groups are met (U.S. Department of Agriculture, 2021). The need for additional research of patch utilization of wild and managed bees has been amplified due to the growing concern that some managed bees, particularly honey bees, may be competing against wild bees for resources (Mallinger et al., 2017; Wojcik et al., 2018). Understanding local habitat use by wild bees and managed honey bees will provide valuable information to resource managers seeking to conserve existing habitats and creating new habitats. Furthermore, studies of patch use by wild and managed bees will elucidate the degree to which these bee groups exhibit differences in habitat requirements. Research on patch utilization of bees can be particularly valuable in agricultural areas of the mid‐western United States, which harbor a majority of U.S. commercial honey bee colonies (U.S. Department of Agriculture, 2019) and provide natural areas for many wild bees, including those of conservation concern (Evans et al., 2018; Lane et al., 2020).

The Prairie Pothole Region (PPR) supports the highest density of honey bee colonies in the United States (Hellerstein et al., 2017), and the number of colonies brought to this region continues to increase. For example, the number of registered honey bee colonies in North Dakota increased from 300,000 in 2000 to 470,000 in 2017 (U.S. Department of Agriculture 2002, 2017, 2017). Concurrent with rising numbers of honey bee colonies, increasing amounts of grassland habitat within the PPR have been converted to corn and soybeans (Lark et al., 2015; Wright & Wimberly, 2013). Grasslands in this region provide important forage sites and refugia for wild bees and honey bees. Specifically, wild bee relative abundance, species diversity, and functional diversity are all higher in areas with larger amounts of natural land covers such as grasslands and wetlands (Evans et al., 2018; Lane et al., 2020; Vickruck et al., 2019). Honey bee colony survival is higher and colony size is larger at apiaries surrounded by more grassland (Smart et al., 2016, 2018). Rising global demand for biofuel feedstocks and commodity crop exports are likely to contribute to continued conversion of grassland to cropland in the PPR (Lark et al., 2015; Wright et al., 2017). Ultimately, managers and policymakers seek strategies for supporting a vibrant beekeeping industry while protecting wild bee populations in a rapidly changing landscape.

We collected wild bee and honey bee detection and nondetection data across grassland transects (i.e., sampling units) within the PPR of North Dakota, South Dakota, and Minnesota to estimate the probability of bee use of transects during the summer and detection probabilities in relation to local weather and the abundance and richness of floral resources occurring in grasslands. We used occupancy models (MacKenzie et al., 2017) to determine how use and frequency of use of transects by wild bees and honey bees were related to the abundance of native and non‐native flowers and the richness of flowers during multiple sampling events throughout the growing season (i.e., June–September). In addition, we tested whether wild bee use or frequency of use of resource patches was related to the distance to commercial apiaries containing >12 honey bee colonies. We investigated dietary niche overlap by quantifying 738 wild bee and 4089 honey bee host–plant interaction records. Through our research, we elucidate habitat factors that influence the frequency by which both species groups use resource patches, and highlight the degree to which honey bees and wild bees exhibit floral resource overlap across the PPR.

## 2 METHODS

### 2.1 Study area

**FIGURE 1:**
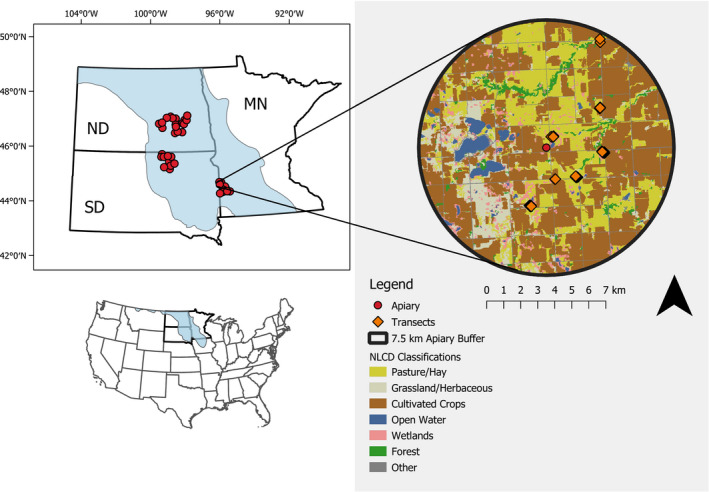
The Prairie Pothole Region of the United States and Canada. Inset image is of a single apiary location (red circle) with a 7.5‐km buffer containing plant and bee transect locations (orange diamond)

Our research occurred in the PPR of North Dakota, South Dakota, and Minnesota in 2016 and 2017 (Figure 1). Although much of the region has been converted to farmland, the PPR still possesses some of the highest densities of wetlands in North America (Lane & D'Amico, 2016) and remnant areas of tall‐grass and mixed‐grass prairie. Estimates are equivocal, but <30% of native grasslands in the Great Plains remain intact and the rate of grassland loss is accelerating (Claassen et al., 2011; Samson et al., 2004; Stephens et al., 2008). Ideal weather conditions along with remnant grasslands, field edges, and wetland buffers that support flowers make the PPR an attractive landscape to beekeepers and their honey bee colonies (Gallant et al., 2014; Otto et al., 2016). Consequently, North Dakota, South Dakota, and Minnesota support approximately 836,000 honey bee colonies annually and are among the top honey‐producing states in the United States (U.S. Department of Agriculture, 2017).

### 2.2 Bee and plant transect searches

This study was part of a larger research project designed to assess the impact of forage availability on honey bee colony health (Smart et al., 2018). First, we randomly selected 36 honey bee apiaries that were managed by collaborating beekeepers and existed across a row‐crop to grassland gradient in the PPR (See Smart et al., 2018 for addition methods on apiary selection). Our study area encompassed approximately 40,000 km^2^ (Figure 1). We selected 239 grassland fields that were within 7.5 km of the selected apiaries distributed throughout the PPR and were located on private or public grasslands such as Conservation Reserve Program (CRP) lands, Environmental Quality Incentives Program (EQIP) lands, managed pasture, hayfields, roadside ditches, Waterfowl Production Areas (WPA), National Wildlife Refuges (NWR), Wildlife Management Areas (WMA), and lands enrolled in the Bee and Butterfly Habitat Fund. Collectively, the fields we selected represented working grasslands managed for hay or grazing, wildlife habitat, and engineered pollinator habitat. Our study did not include managed prairie preserves that have been shown to harbor a high diversity flowers and wild bees in our region (Lane et al., 2020), but are nonetheless uncommon in our study region. The local landscape surrounding our fields generally consisted of a heterogeneous mix of corn, soybeans, and small grains, intermixed with patches of managed grassland and isolated wetlands. We obtained landowner permission to survey 1048 transects in 2016 and resampled 386 of these transects in 2017. In 2017, we added 60 new transects consisting of first‐year pollinator habitat plantings for a total of 1108 transects sampled in 2016 and 2017. Within most fields, we randomly selected one to eight transects, with larger fields having more transects. We had two fields >2.6 km^2^ where we randomly selected 11 and 22 transects due to their large size. The mean distance to the nearest transect was ~60 m (range 10–450 m). Over 95% of the 1108 transects were located within 5 km of known apiary sites, with only 36 transects more than 5 km from an apiary. Locating transects within this distance ensured high potential for use by honey bees.

Each 2 × 20 m sampling unit (transect) was surveyed during three time periods (08 June–15 July, 16 July–15 Aug, 16 Aug–15 Sept) during the growing season. Whenever possible, each transect was surveyed every 30 days. Surveys consisted of a single observer who quantified floral resources within the transect boundary and noted observations of honey bees and wild bees collecting pollen and nectar from flowers therein. Transect boundaries were delineated with a meter tape laid along the center line and metal flags at the four corners. At the onset of each survey, the observer recorded wind speed, relative humidity, and air temperature with a Kestrel 3000 Pocket Weather Station and visually estimate percent cloud cover. Observers did not conduct surveys during rain, wind speeds >40 kph, or temperatures below 15℃. Exceptions include a subset of August to September surveys (2%) where time restrictions and long bouts of cold weather required observers to conduct surveys in temperatures ranging from 10 to 15℃. During each survey, observers moved systematically and slowly along each transect, counting the number of forb basal stems that supported one or more inflorescences (i.e., ramets), which served as our index of flower abundance (hereafter referred to as flower abundance). Flowering plants were identified to species in most cases. Bees flying through the transect, but not landing on a flower, were not recorded. Each survey took an average of 4 ± 3 (i.e., ±1 SD) minutes to complete.

### 2.3 Distance to apiary

Although our study was not designed to directly test competitive interactions between managed honey bees and wild bees, the abundance of honey bee colonies in our study landscape provided us with an opportunity to determine whether wild bee patch utilization was related to the distance to honey bee colonies. Researchers have used distance to nearest honey bee apiary as a proxy variable to measure the potential effects of foraging honey bees on floral resources and wild bee local abundance (Henry & Rodet, 2018, 2020; Hudewenz & Klein, 2013). Because one of our initial selection criteria was to have all transects located within 7.5 km of known apiary locations, calculating the linear distance between transects and apiaries was straightforward. We also used existing apiary registration data in North Dakota and South Dakota to verify the shortest distance between apiaries and transects. We did this because there were a few cases where our focal apiary used in initial site selection was not the closest apiary to a transect. We obtained apiary registration data from the state department of agriculture in North Dakota (https://www.nd.gov/ndda/) and South Dakota (https://sdda.sd.gov/ag‐services/). The North Dakota and South Dakota state Departments of Agriculture require beekeepers to register the physical locations of their apiaries prior to placing hives at an apiary. Apiary location data are managed by the state and are publicly available upon request. We ensured registered apiaries contained honey bee colonies by locating colonies on 2014–2017 aerial photographs via Google Earth^®^. Aerial photograph interpretation was done by technicians at the University of North Dakota and Northern Prairie Wildlife Research Center in 2017 and 2018. Minnesota does not require beekeepers to register apiary locations, so we obtained Minnesota apiary location data from Adee Honey Farms, the company that manages a majority of the honey bee colonies within our Minnesota study region. We verified these apiary locations on aerial photographs and checked to ensure there were no other apiaries within proximity to our transect locations. We then calculated the linear distance between each transect sampling unit and the nearest apiary.

### 2.4 Flower visitations

We summarize wild bee and honey bee flower visitation data by calculating the most commonly visited native and non‐native forbs. We report proportions of native and non‐native flower utilization by wild bees and honey bees. All associated data for this research are available as a USGS data release (https://doi.org/10.5066/P9O61BCB).

### 2.5 Single‐season, single‐species occupancy models

Occupancy models (MacKenzie et al., 2017) have been widely used by ecologists to model species distribution patterns and fine‐scale habitat associations (Bailey et al., 2009; McCune et al., 2020) and are increasingly used by multiple state, federal, and citizen science monitoring programs. Occupancy models use detection–nondetection data collected across multiple surveys of selected sampling units to estimate the probability that a species occupies, or uses, a unit while accounting for imperfect detection (i.e., false absences). While broadly applied in vertebrate systems, occupancy models have only recently been used in studies of bees (Graves et al., 2020; Landsman et al., 2019; McCune et al., 2020). These studies suggest bee detection probability is not perfect and varies with weather, time of year, and sampling effort, thereby highlighting the need to account for imperfect detection. While many arthropod and pollinator studies commonly refer to species “occurrence” as a primary response variable (Seibold et al., 2019), most do not account for imperfect detection and thus the two processes, occurrence and detection, are confounded. Occupancy models provide a useful framework for obtaining robust estimates of species distribution patterns and understanding the association between patch utilization and local habitat variables when detection probability varies among patches due to flower or bee abundance or among surveys due to local weather conditions, survey effort, or observer experience.

Although our study was not originally designed within an occupancy framework, we used the three replicate surveys to estimate occupancy and detection probability for two different groups of bees (honey bees and wild bees) at transects within our study area. We pooled data for all wild bees because the detections of individual species or genera were too low to permit species‐specific analyses and identifying species of wild bees without in vitro techniques was not possible. Although lower taxonomic identification of wild bees may have improved our inferences, we note that wild bees are often managed as a comprehensive group, and species‐specific management is not employed except for agriculturally important species (e.g., *Apis mellifera*) or species of conservation concern (e.g., *Bombus affinis*).

Based on our sampling design, occupancy (denoted Ψ) represents the probability that a transect (sampling unit) is used by the target group during the 3.5‐month growing season. Interpreting occupancy as “use” is common for studies where individuals may move in and out of the sampling unit during the season but may not always be present at the unit during a given survey (MacKenzie et al., 2017, p. 147). This seems likely in our system, as a group of bees may use a transect during the growing season but may not always be present at the transect during a given survey. Detection probability (denoted *p*) represents the probability that an individual of the target group is detected on a survey, given the transect is used during the season. This conditional detection probability is the product of two different components: The probability an individual of the target group was present in the transect at the time of the survey and the probability the target group was detected, given it was present. The covariates we considered could influence either of these detection components; for example, wind speed and cloud cover likely impact the ability of observers to detect bees given they are present in the transect. Other detection covariates are more closely related to bee flight activity (temperature and humidity) or frequency of use of a transect (i.e., flower abundance and richness), which affects the probability bees are present at the time of the survey. We followed guidance from MacKenzie et al. (2017, p. 147) and interpreted detection probability as the relative frequency of use by wild or honey bees during the growing season, as we believe variation in this component contributed more to the variation in detection probability in our system.

We conducted separate occupancy analyses for honey bees and wild bees. Prior to analysis, we removed all transects where no flowers were detected within the growing season.

We generated detection histories of honey bees and wild bees at each remaining transect using our three surveys and used this data to explore factors influencing bee use and frequency of use (detection probability) at transects in our study area (see next section). We assumed our target groups (i.e., honey bee and wild bees) used transects within the growing season in a random manner (i.e., individuals entered or left the unit randomly during the course the growing season). We also assumed the detection histories we collected at selected units were independent, and we tested this assumption via a goodness‐of‐fit test (see below, MacKenzie & Bailey, 2004).

### 2.6 Covariate explanation and predictions

We predicted wild bee transect use and frequency of use would be positively related to flower richness (*Richness*) and abundance of native plants (*Native*), based on previous research highlighting wild bee preference of native flowers (Morandin & Kremen, 2013). However, non‐native flowers can also be important to wild bees (Williams et al., 2011) but likely not to the degree that native flowers are important. We tested this by treating native and non‐native (*Non*‐*native*) flower abundance as separate covariates and compared them to models where both native and non‐native flower abundance were combined as a single covariate (*All_Flowers*). When modeling use, flower abundance was the total number of flowers at transect unit *i*, summed across the three surveys. Likewise, flower richness represented the number of unique flower species detected at transect unit *i*, during the three surveys. When modeling frequency of use (detection probability), flower abundance represented the number flowers at transect unit *i*, during survey *j*, while flower richness represented the number of unique flower species detected at transect unit *i*, during survey *j*. We tested whether wild bee use and frequency of use were lower at transects that were closer to commercial apiaries by including distance to apiary as a covariate in our models (*Distance*). All flower abundance and distance to apiary covariates were divided by 1000 so that parameter estimates reflected the same magnitude of change (e.g., per 1000 flowers or per 1000 m). Although understanding the relationship between local weather and bee frequency of use was not our primary objective, we predicted wild bee frequency of use would be negatively related to wind speed (*Wind*) and would increase during warm (*Temp*) and humid (*Humidity*) days, as these factors may influence bee foraging activity.

Honey bee foragers can share information on the availability of floral resources among members within in a colony, which allows them to quickly extract resources from dense flower patches (Seeley, 1995). Therefore, we predicted honey bee transect use and frequency of use would be more closely related to total flower abundance, regardless of whether the flowers were native or non‐native. Honey bees can travel several kilometers in search of resources; however, their primary foraging distance is <1 km from the hive (Seeley, 1995). We expected transects further from known apiaries would be visited less frequently by honey bees. Because honey bees use the angle of the sun to navigate and share information on the availability of resources among colony members, we predicted honey bee frequency of use would be lower during cloudy (*Cloud*) and cooler days. Similar to wild bees, we predicted honey bee frequency of use would decrease during windy and less humid days as these factors may influence bee foraging activity.

### 2.7 Occupancy model building

We tested for collinearity between all occupancy and detection covariates using a Pearson's product–moment correlation (Sokal & Rohlf, 1981). We did not include covariates with correlation coefficient ≥0.7 in the same model. To account for potential overdispersion and lack of independence, we conducted a goodness‐of‐fit test (GOF; MacKenzie & Bailey, 2004) using the global model for each group of bees: Ψ (Native + Non‐native + Richness + Distance), *p*(Native*_j_* + Non‐native*_j_* + Richness*_j_* + Temperature^2^ + Wind + Distance). If overdispersion existed, we based model selection on quasi‐Akaike's information criterion (QAIC; (Burnham & Anderson, 2002)) and adjusted measures of precision according to the estimated overdispersion parameter ($\hat{c}$).

Given our large number of weather and habitat covariates, we used a step‐wise approach to model building and fit all models using the package Unmarked (Fiske & Chandler, 2011) in R (R Core Team, 2017). We started with a general model structure that included the effects of native and non‐native flower abundance, flower richness, and distance to nearest apiary on bee use and detection (i.e., frequency of use), Ψ(Native + Non‐Native + Richness + Distance), *p*(Native*_j_* + Non‐native*_j_* + Richness*_j_* + Distance). To this general structure, we first considered additive combinations of weather covariates (e.g., wind speed, temperature, etc.) to determine their influence on wild bee and honey bee frequency of use (Table 3 [honey bee] and 6 [wild bee]). Preliminary analysis indicated a quadratic relationship between bee detections and temperature (i.e., *Temp* + *Temp^2^*), so we considered only the quadratic form in our models. Retaining the best‐supported weather covariates for each group of bees, we explored how frequency of use varied with floral resources and distance to nearest apiary. Specifically, we fit structures with additive combinations of the three floral covariates both with and without distance to apiary (i.e., *Distance*), and a model with the combined flower abundance observed during each survey (*All_Flowers_j_*; Table 4 (honey bee) and 7 (wild bee)). Finally, we used the best‐supported detection (i.e., frequency of use) structure for each group of bees to determine the effects of seasonal flower abundance, flower richness, and distance to nearest apiary on the probability a transect was used during the growing season. We considered occupancy (use) structures that included all additive combinations of our three floral resource covariates or the combined abundance of all flowers, and models with and without distance to nearest apiary (Table 5 (honey bee) and 8 (wild bee)). During each initial step in the model building process, we include a null model (Ψ (.) or *p*(.)), but generally there was little support for the null model. We used Akaike's information criterion (AIC or QAIC) and model weights to rank candidate models (Burnham & Anderson, 2002) and report parameter estimates and standard errors for supported models. We identified uninformative parameters or “pretending variables” by comparing log‐likelihood or deviance values for models with different number of parameters and examining estimates and 95% confidence intervals for associated model parameters (Arnold, 2010).

## 3 RESULTS

### 3.1 Flower and weather covariates, and flower visitations

In general, native and non‐native flower abundances were highest during our first survey (June through mid‐July) and declined thereafter (Table 1). Flower richness was highest during our second survey (mid‐July through mid‐August). The transect‐level covariates *All Flowers* and *Non*‐*Native Flowers* were highly correlated (Table 2) and not used in the same parameter structure. All other covariates were not strongly correlated (*r* < .7; Table 2).

**TABLE 1:** Mean covariate values (±1 SD) used in wild bee and honey bee occupancy models

Covariate	Early	Mid	Late	All season
Temperature (°C)	25.5 ± 3.6	27.5 ± 4	23.8 ± 5.4	NA
Cloud Cover (%)	31.1 ± 33.9	32.1 ± 34.6	33.2 ± 37.6	NA
Wind Speed (kph)	8.5 ± 7.6	8.1 ± 7.1	8.2 ± 7.5	NA
Humidity (%)	55.5 ± 12.1	61.9 ± 13.8	59.2 ± 13.7	NA
Native Flowers	38.7 ± 99.6	29.02 ± 65.3	24.9 ± 53.5	90.2 ± 158.3
Non‐Native Flowers	173.6 ± 375.2	103.4 ± 225.6	60.5 ± 155.2	329.7 ± 552.7
Flower Richness	3.0 ± 2.6	3.6 ± 3	2.8 ± 2.4	6.4 ± 4.3
All Flowers	214.0 ± 386.8	132.3 ± 234.5	85.4 ± 163.8	420.0 ± 573.8
Distance (m)	1805 ± 1383	1805 ± 1383	1805 ± 1383	1805 ± 1383

Note

Early, mid‐, and late corresponded to surveys conducted in 08 June–15 July, 16 July–15 August, and 16 August–15 September, respectively, in the Prairie Pothole Region of the United States and Canada.

**TABLE 2:** Correlation coefficient (*r*) matrix of survey and transect covariates used in wild bee and honey bee occupancy models

Survey covariates	Temperature	Cloud cover	Wind speed	Humidity	Native flowers	Non‐native flowers	Flower richness	All flowers	distance
Temperature	1	−0.335	−0.147	−0.326	0.044	−0.024	0.007	−0.011	−0.053
Cloud cover		1	−0.08	0.424	−0.006	0.045	0.055	0.041	0.018
Wind speed			1	−0.001	−0.099	−0.023	−0.136	−0.049	0.221
Humidity				1	−0.026	−0.007	0.032	−0.014	−0.029
Native flowers					1	0.005	0.496	0.282	−0.035
Non‐native flowers						1	0.236	**0.961**	0.034
Flower richness							1	0.363	−0.096
All flowers								1	0.024
Distance									1

Site covariates	All flowers	Native flowers	Non‐native flowers	Flower richness	Distance
All flowers	1	0.276	**0.96**	0.353	0.040
Native flowers		1	−0.005	0.63	−0.044
Non‐native flowers			1	0.192	0.054
Flower richness				1	−0.122
Distance					1

Note

Highly correlated covariates are in bold.

**FIGURE 2:**
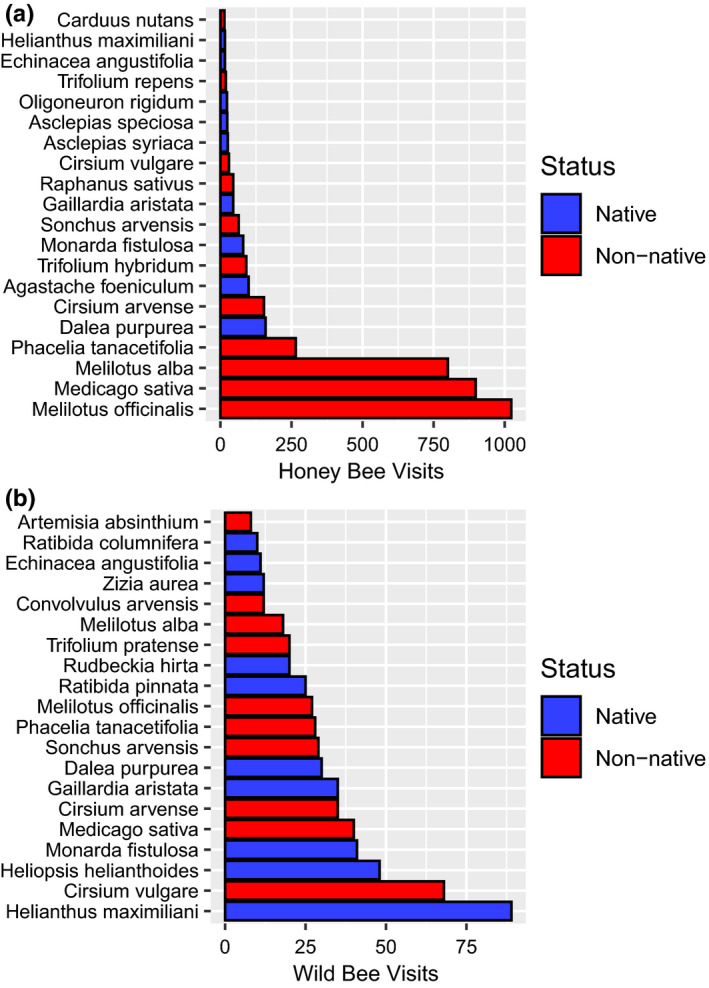
Flower visitations of honey bees (a) and wild bees (b) detected on 1030 transects conducted in North Dakota, South Dakota, and Minnesota, USA, in 2016 and 2017. Only the top 20 visited plants are shown for clarity

*Medicago sativa* (alfalfa [non‐native], 43% of total), *Melilotus officinalis* (yellow sweet clover [non‐native], 14% of total), *Melilotus alba* (white sweet clover [non‐native], 6%), *Rudbeckia hirta* (black‐eyed Susan [native], 4%), and *Medicago lupulina* (black medic [non‐native], 2%) were the five most common flowers on our transects. Honey bees and wild bees were observed on 65 and 68, respectively, of the 228 flower species detected along transects. Across all transects and surveys, we observed 4039 and 738 honey bee and wild bee flower visitations, respectively. The top five plant species visited by honey bees were *M*. *officinalis* (25%, non‐native), *M*. *sativa* (22%, non‐native), *M*. *alba* (20%, non‐native), *Phacelia tanacetifolia* (lacy phacelia, 6%, non‐native), *Dalea purpurea* (purple prairie clover, 4%, native), representing 77% of all honey bee detections (Figure 2a). Across all honey bee flower visitations, 85% were on non‐native flowers. The top five plant species visited by wild bees were *Helianthus maximiliani* (Maximilian sunflower [native], 12%), *Cirsium vulgare* (bull thistle [non‐native], 9%), *Heliopsis helianthoides* (smooth oxeye [native], 7%), *Monarda fistulosa* (wild bergamot [native], 6%), and *M*. *sativa* (non‐native, 6%), representing 40% of all wild bee detections (Figure 2b). Across all wild bee visitations, 43% were on non‐native flowers.

### 3.2 Honey bee probability of use and detection

Prior to modeling, we eliminated 78 of the 1108 transects because no flowers were detected throughout a growing season (final number of transects = 1030). Across our survey replicates, honey bees were detected at least once at 490 of 1030 transects. The goodness‐of‐fit test of our global model revealed evidence of lack of fit (χ^2^ = 41.04 *p* = .02, $\hat{c}$ = 2.7). Initially, we thought the lack of fit could be due to a lack of independence between our 2016 and 2017 transects; however, the lack of fit persisted when we ran the GOF test on just the 2016 data. Visual inspection of the observed and expected frequencies for the observed detection histories revealed a large contribution to the chi‐square value was attributed to a few detection histories from transects that lacked all three surveys. These detection histories had extremely low expected values (i.e., *E_h_* < 2), resulting in large chi‐square values, which is a known problem with this goodness‐of‐fit test (MacKenzie et al., 2017, p. 161). There was good agreement between observed and expected frequencies for transects with all three surveys (i.e., no missing values); still, we used quasi‐AIC (QAIC) model selection and inflated the standard errors using the estimate overdispersion parameter ( for our honey bee analysis.

**FIGURE 3:**
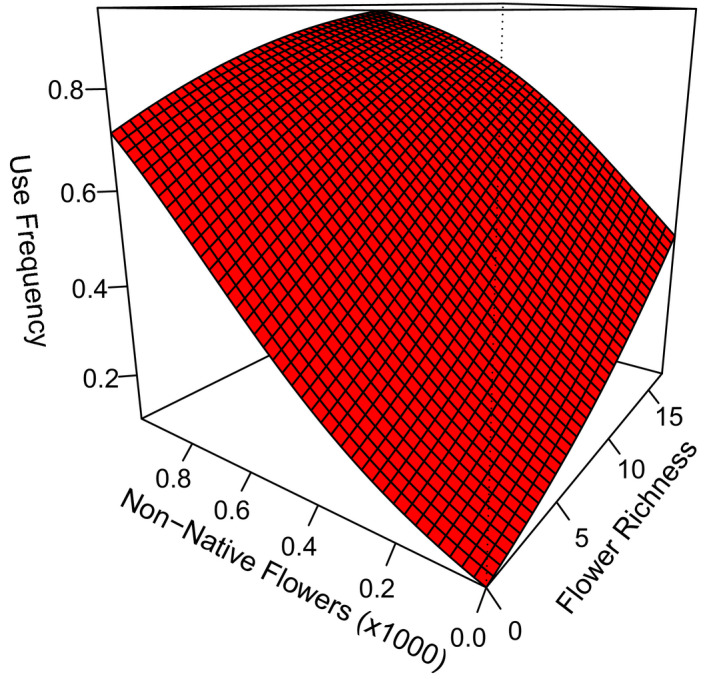
Honey bee frequency of use (detection probability) modeled as a function of the total number of non‐native flowers and flower richness during a survey along 2 × 20 m grassland transects in North Dakota, South Dakota, and Minnesota, USA, in 2016 and 2017. Estimates were based on the best‐supported model Ψ (non‐native + native + richness), *p*(distance + non‐native*_j_* + native*_j_* + richness*_j_* temp^2^ *_j_* + cloud*_j_* + humidity*_j_*), using the mean value for the other covariates. Covariate axes were truncated to show most of our data points (Table 1). Single covariate graphics can be found in Figure A1

Honey bee detection probability, or frequency of use, showed a strong quadratic relationship with temperature (Table 3), initially increasing with air temperature, but plateaued, and decreasing on exceptionally hot days (${\hat{\beta }}_{\mathrm{temp}}$ = 0.41 ± 0.18, ${\hat{\beta }}_{temp^{2}}$ = −0.29 ± 0.13). There was also some evidence that cloud cover and humidity influenced honey bee frequency of use, but confidence intervals of these weather covariates overlapped zero after adjusting for overdispersion (Table 3). Accounting for variation associated with these weather covariates, we investigated the role of floral resource abundance, richness, and distance to nearest apiary on honey bee detection probability and frequency of use (Table 4). Honey bee frequency of use was higher at times and transects with higher abundance of non‐native flowers and higher richness of flowering species (Figure 3, Table 4). There was some evidence that transects with higher abundances of native flowers (${\hat{\beta }}_{native_{j}}$ = 4.14 ± 2.32) were more frequently used by honey bees; however, the standard error associated with the native flower parameter estimate was large, suggesting uncertainty regarding the strength of this relationship (Table 4). Not surprisingly, honey bee frequency of use declined with distance from apiaries (${\hat{\beta }}_{\mathrm{distance}}$ = −0.29 ± 0.13). Collectively, our results suggest that honey bees were more frequently detected at times/transects that had either increased flower richness or higher abundance of non‐native flowers (Figure 3) and less likely detected at transects that were further from apiaries.

**TABLE 3:** Quasi‐Akaike's information criterion (QAIC) ranking of candidate models investigating the effect of survey‐specific weather variables on honey bee detection along 1030, 2 × 20 m transects in North Dakota, South Dakota, and Minnesota, USA, in 2016 to 2017. *K* is the number of model parameters, ΔQAIC is difference from top model, w is model weight, QDeviance is −2Log(*L*)/$\hat{c}$ (i.e., adjustment for overdispersion, $\hat{c}$ = 2.7)

Model	*K*	QAIC	ΔQAIC	*w*	QDeviance	*p*_cloud_	*p*_humidity_	*p*_temp_	*p* temp 2	*p*_wind_
*p* temp + temp 2 _+ cloud + humid_	15	978.5	0.0	0.43	−948.5	0.09 ± 0.16	0.02 ± 0.16	0.41 ± 0.18	−0.29 ± 0.13	
*p* temp + temp 2 _+ humid + wind_	15	978.6	0.1	0.41	−948.6		0.02 ± 0.15	0.30 ± 0.18	−0.28 ± 0.13	−0.54 ± 0.25
*p* temp + temp 2 _+ cloud + humidity + wind_ ^a^	16	980.5	2.0	0.16	−948.5	0.06 ± 0.17	0.002 ± 0.17	0.30 ± 0.18	−0.28 ± 0.13	−0.54 ± 0.22^a^
*p* temp + temp 2	13	994.8	16.3	0.00	−968.8					
*p* temp + temp 2 _+ cloud_	14	995.7	17.2	0.00	−967.7					
*p* temp + temp 2 _+ humidity_	14	996.4	17.9	0.00	−968.4					
*p*_wind_	12	997.1	18.6	0.00	−973.1					
*p*_humidity + wind_	13	997.4	18.9	0.00	−971.4					
*p* temp + temp 2 _+ cloud + humidity_	15	997.6	19.1	0.00	−967.6					
*p*_cloud + wind_	13	997.9	19.4	0.00	−971.9					
*p* temp + temp 2 _+ wind_	14	1003.2	24.7	0.00	−975.2					
*p*_(.)_	11	1023.1	44.6	0.00	−1001.1					
*p*_humidity_	12	1023.3	44.9	0.00	−999.3					
*p*_cloud_	12	1024.5	46.0	0.00	−1000.5					
*p*_cloud + humidity_	13	1025.3	46.8	0.00	−999.3					

Note

Estimated effect sizes (beta estimates ± 1SE) are listed for models with ΔQAIC < 10. All models include the general structure Ψ (non‐native + native + richness + distance), *p*(non‐native*_j_* + native*_j_* + richness*_j_* + distance + weather structure in model name below).

^a^ Pretending variable (Arnold, 2010)

**TABLE 4:** Quasi‐Akaike's information criterion (QAIC) ranking of candidate models investigating the effect of survey‐specific flower abundance and richness variables on honey bee detection along 1030, 2 × 20 m transects in North Dakota, South Dakota and Minnesota, USA, in 2016 to 2017

Model:	*K*	QAIC	ΔQAIC	*w*	QDeviance	*p*_all flowers_	*p*_native_	*p*_non‐native_	*p*_richness_	*p*_distance_
*p*_native + non‐native + richness + distance_	15	997.6	0.0	0.53	−967.6		4.14 ± 2.32	2.02 ± 0.51	0.18 ± 0.06	−0.29 ± 0.13
*p*_all flowers + richness + distance_	14	997.9	0.3	0.46	−969.9	2.05 ± 0.52			0.21 ± 0.06	−0.29 ± 0.13
*p*_non‐native + richness + distance_	14	1005.4	7.8	0.01	−977.4			1.86 ± 0.06	0.24 ± 0.06	−0.29 ± 0.13
*p*_non‐native + native + richness_	14	1010.9	13.2	0.00	−982.9					
*p*_all flowers + richness_	13	1011.2	13.6	0.00	−985.2					
*p*_non‐native + richness_	13	1018.0	20.4	0.00	−992.0					
*p*_native + non‐native + distance_	14	1019.5	21.9	0.00	−991.5					
*p*_native + non‐native_	13	1036.7	39.0	0.00	−1010.7					
*p*_all flowers_	12	1036.9	39.3	0.00	−1012.9					
*p*_all flowers + distance_	13	1038.0	40.4	0.00	−1012.0					
*p*_native + richness + distance_	14	1048.9	51.3	0.00	−1020.9					
*p*_richness + distance_	13	1051.5	53.8	0.00	−1025.5					
*p*_native + richness_	13	1056.4	58.7	0.00	−1030.4					
*p*_richness_	12	1058.7	61.1	0.00	−1034.7					
*p*_non‐native_	12	1083.5	85.9	0.00	−1059.5					
*p*_native + distance_	13	1084.5	86.9	0.00	−1058.5					
*p*_non‐native + distance_	13	1085.0	87.4	0.00	−1059.0					
*p*_native_	12	1094.0	96.4	0.00	−1070.0					
*p*_distance_	12	1117.2	119.6	0.00	−1093.2					
*p*_(.)_	11	1127.0	129.4	0.00	−1105.0					

Note

*K* is the number of model parameters, ΔQAIC is difference from top model, w model weight, QDeviance is −2Log(*L*)/$\hat{c}$ (i.e., adjustment for overdispersion, $\hat{c}$ = 2.7). Estimated effect sizes (beta estimates ± 1SE) are listed for models with ΔQAIC < 10. All models include the structure Ψ (non‐native + native+ richness), *p*(temp^2^ *_j_* + cloud*_j_* + humidity*_j_* + survey‐specific resource variables listed in the model name below).

**FIGURE 4:**
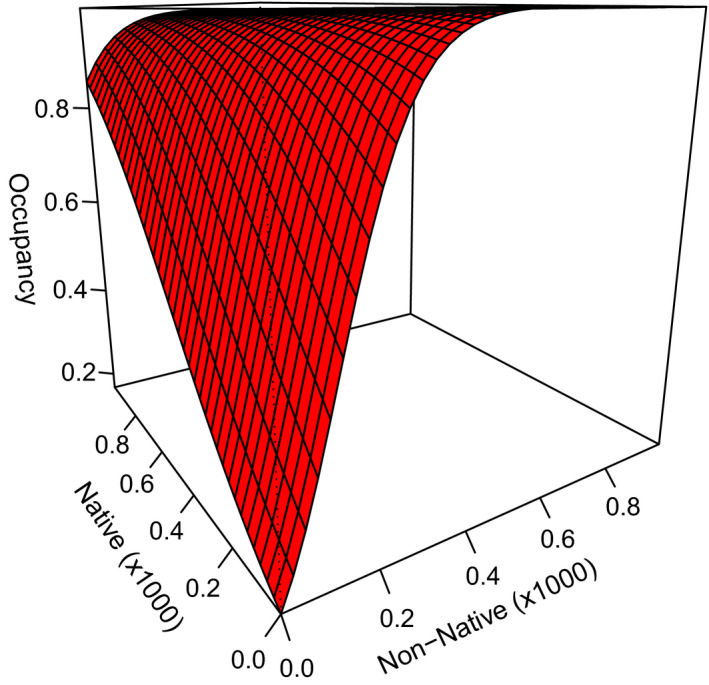
Honey bee occupancy estimates (i.e., probability of use) as a function of the abundance of non‐native and native flowers counted along 2 × 20 m grassland transects in North Dakota, South Dakota, and Minnesota, USA, in 2016 and 2017. Estimates were based on the best‐supported model Ψ (on‐native + Native), *p*(distance + non‐native*_j_* + native*_j_* + richness*_j_* temp^2^ *_j_* + cloud*_j_* + humidity*_j_*), using the mean value for the other covariates. Covariate axes were truncated to show most of our data points (Table 1). Single covariate graphics can be found in Figure A2

We used the most parsimonious detection structure to investigate factors that influenced the likelihood that transects were used at least once by honey bees during the growing season (Table 5). The probability that a transect was used by honey bees increased with non‐native (${\hat{\beta }}_{non‐nat}$ = 13.8 ± 6.3) and native (${\hat{\beta }}_{\mathrm{native}}$ = 3.4 ± 2.1) flower abundance. The estimated effect of non‐native flowers was four times higher than for native flowers, suggesting that honey bee use was much greater at transects with <1000 non‐native flowers than it was for transects with <1000 native flowers (Figure 4). However, at transects with >1000 flowers, honey bee probability of use approached 1.0 regardless of flower indigenous status. Although a model with an additive effect of flower richness had some support (*w* = 0.23, Table 5), this was a pretending variable (Arnold, 2010), suggesting there was no relationship between honey bee use and flower richness (${\hat{\beta }}_{\mathrm{richness}}$ = −0.02 ± 0.1). We found no evidence that distance to nearest apiary affected honey bee use (Table 5), suggesting honey bees were equally likely to use transects within 7.5 km of an apiary, at least once during the growing season.

**TABLE 5:** Quasi‐Akaike's information criterion (QAIC) ranking of candidate models investigating the effect of seasonal flower abundance and richness variables on honey bee use of 1030, 2 × 20 m transects in North Dakota, South Dakota, and Minnesota, USA, in 2016–2017

Model	*K*	QAIC	ΔQAIC	*w*	QDeviance	Ψ_all flowers_	Ψ_native_	Ψ_non‐native_	Ψ_richness_	Ψ_distance_
Ψ_non‐native + native_	13	994.1	0.0	0.61	−484.1		3.4 ± 2.1	13.8 ± 6.3		
Ψ_non‐native + native + richness_ ^a^	14	996.0	1.9	0.23	−484.0		3.8 ± 2.7	14.0 ± 6.5	−0.02 ± 0.10^a^	
Ψ_non‐native + native + richness_ ^a^_+ distance_ ^a^	15	997.6	3.5	0.10	−483.8		3.8 ± 2.8	14.4 ± 6.8	−0.02 ± 0.10^a^	0.15 ± 0.42^a^
Ψ_non‐native + richness_	13	1001.2	7.1	0.02	−487.6			11.8 ± 5.5	0.09 ± 0.15	
Ψ_all flowers + richness_	13	1002.0	7.9	0.01	−488.0	12.7 ± 6.9			−0.13 ± 0.12	
Ψ_non‐native + richness + distance_	14	1002.7	8.6	0.01	−487.3			12.0 ± 5.5	0.08 ± 0.08	0.18 ± 0.43
Ψ_non‐native_	12	1002.8	8.6	0.01	−489.4			11.7 ± 5.5		
Ψ_all flowers_	12	1003.6	9.5	0.01	−489.8	9.9 ± 4.9				
Ψ_all flowers + richness + distance_	14	1003.8	9.7	0.00	−487.9	13.3 ± 8.0			−0.14 ± 0.13	0.18 ± 0.43
Ψ_distance_	12	1015.5	21.4	0.00	−495.8					
Ψ_richness + distance_	13	1017.2	23.1	0.00	−495.6					
Ψ_native + distance_	13	1017.5	23.4	0.00	−495.7					
Ψ_all flowers + distance_	13	1017.5	23.4	0.00	−495.8					
Ψ_non‐native + distance_	13	1017.5	23.4	0.00	−495.8					
Ψ_native + richness + distance_	14	1019.1	25.0	0.00	−495.5					
Ψ_native + non‐native + distance_	14	1019.5	25.4	0.00	−495.7					
Ψ_(.)_	11	1020.5	26.4	0.00	−499.2					
Ψ_richness_	12	1022.3	28.1	0.00	−499.1					
Ψ_native_	12	1022.5	28.4	0.00	−499.2					
Ψ_native + richness_	13	1024.2	30.1	0.00	−499.1					

Note

*K* is the number of model parameters, ΔQAIC is difference from top model, w is model weight, QDeviance is −2Log(*L*)/$\hat{c}$ (i.e., adjustment for overdispersion, $\hat{c}$ = 2.7). Estimated effect sizes (beta estimates ±1SE) are listed for models with ΔQAIC < 10. All models include the best‐supported detection structure: *p*(distance + native*_j_* + non‐native*_j_* + richness*_j_* + temp^2^ *_j_* + cloud*_j_* + humidity*_j_*).

^a^ Pretending variable (Arnold, 2010).

### 3.3 Wild bee probability of use and detection

**FIGURE 5:**
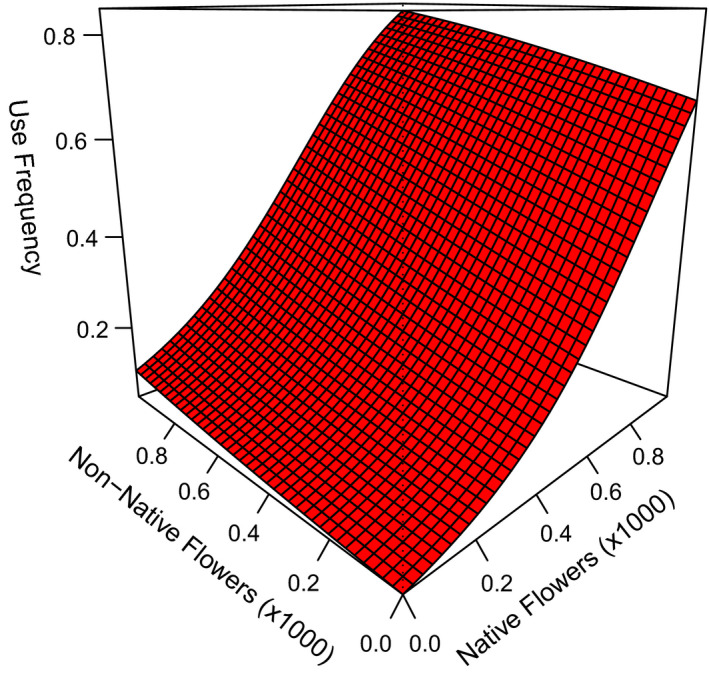
Wild bee frequency of use (detection probability) explained as a function of the number of native and non‐native flowers counted during a single survey along 2 × 20 m grassland transects in North Dakota, South Dakota, and Minnesota, USA, in 2016 and 2017. Estimates were based on the best‐supported model Ψ (non‐native + native + richness), *p*(non‐native*_j_* + native*_j_* + richness*_j_* + temperature2 + wind), using the mean value for the other covariates. Covariate axes were truncated to show most of our data points (Table 1). Single covariate graphics can be found in Figure A3

We detected wild bees at least once at 266 of 1030 transects. We detected honey bees at 89 of the 266 transects where wild bees were detected. We found no evidence of lack of fit based on the GOF test (χ^2^ = 15.9 *p* = .38, $\hat{c}$ = 1.0) and therefore used AIC for model selection. Wild bee detection probability decreased with increasing relative humidity, temperature, and wind speed (Table 6). Accounting for variation in wild bee detection due to weather variables, we found positive relationships between wild bee frequency of use and the abundance of native flowers (${\hat{\beta }}_{native_{j}}$ = 3.9 ± 0.65), non‐native flowers (${\hat{\beta }}_{non‐nat_{j}}$ = 0.99 ± 0.17), and flower richness (${\hat{\beta }}_{richness_{j}}$ = 0.24 ± 0.02; Table 7). The estimated effect of native flowers was four times higher than for non‐native flowers for the most supported model (Table 7), suggesting that wild bees used transects with abundant native flowers more frequently than transects with similar abundances of non‐native flowers (Figure 5). The top‐ranking model did not include the distance to nearest apiary; however, the second most supported model suggested wild bees more frequently used transects in close proximity to a honey bee apiary (${\hat{\beta }}_{\mathrm{distance}}$ = −0.12 ± 0.06), but the confidence interval nearly overlapped zero. Collectively, our results suggest the frequency of wild bee use was strongly related to native flower abundance and to a lesser extent to flower richness and non‐native flower abundance.

**TABLE 6:** Akaike's information criterion (AIC) ranking of candidate models investigating the effect of survey‐specific weather variables on wild bee detection along 1030, 2 × 20 m transects in North Dakota, South Dakota, and Minnesota, USA, in 2016 to 2017

Model	*K*	AIC	ΔAIC	*w*	−2LL	*p*_cloud_	*p*_humidity_	*p*_temp_	*p* temp 2	*p*_wind_
*p* temp + temp 2 + humidity + wind	14	1749.3	0.0	0.58	1721.3		−0.25 ± 0.07	−0.20 ± 0.07	−0.06 ± 0.05	−0.58 ± 0.11
*p* temp + temp 2 + cloud + humidity + wind	15	1750.3	1.0	0.34	1720.3	−0.08 ± 0.08	−0.22 ± 0.08	−0.21 ± 0.08	−0.05 ± 0.05	−0.58 ± 0.11
*p*_humidity + wind_	12	1754.0	4.7	0.05	1730.0		−0.25 ± 0.07			−0.55 ± 0.11
*p* temp + temp2 + cloud + wind	14	1756.7	7.4	0.01	1728.7	−0.18 ± 0.7		−0.16 ± 0.07	−0.06 ± 0.05	−0.58 ± 0.11
*p* temp + temp2 + wind	13	1759.4	10.1	0.00	1733.4					
*p* cloud + wind	12	1759.5	10.2	0.00	1735.5					
*p* wind	11	1760.2	10.9	0.00	1738.2					
*p*_humidity_	11	1781.8	32.5	0.00	1759.8					
*p* temp + temp2 + humidity	13	1782.3	33.0	0.00	1756.3					
*p* temp + temp2 + cloud + humidity	14	1783.5	34.2	0.00	1755.5					
*p* cloud + humidity	12	1783.5	34.3	0.00	1759.5					
*p*_cloud_	11	1787.0	37.7	0.00	1765.0					
*p*_(.)_	10	1788.5	39.2	0.00	1768.5					
*p* temp + temp2 + cloud	13	1797.6	48.3	0.00	1771.6					
*p* temp + temp 2	12	1798.2	48.9	0.00	1774.2					

Note

*K* is the number of model parameters, ΔAIC is difference from top model, w model weight, −2LL is the −2*log‐likelihood. Estimated effect sizes (beta estimates ± 1SE) are listed for models with ΔAIC < 10. All models include the structure ψ(non‐native + native + richness + distance), *p*(non‐native*_j_* + native*_j_* + richness*_j_* + distance + weather structure in model name below).

**TABLE 7:** Akaike's information criterion (AIC) ranking of candidate models investigating the effect of survey‐specific flower abundance, richness, and distance to nearest honey bee apiary on wild bee detection along 1030, 2 × 20 m transects in North Dakota, South Dakota, and Minnesota, USA, in 2016 to 2017

Model	*K*	AIC	ΔAIC	*w*	−2LL	*p*_all flowers_	*p*_distance_	*p*_native_	*p*_non‐native_	*p*_richness_
*p*_non‐native + native + richness_	13	1746.5	0.0	0.80	1720.5			3.9 ± 0.65	0.99 ± 0.17	0.24 ± 0.02
*p*_distance + native + non‐native + richness_	14	1749.3	2.8	0.20	1721.3		−0.12 ± 0.06	4.2 ± 0.70	0.89 ± 0.16	0.28 ± 0.02
*p*_distance + all flowers + richness_	13	1763.8	17.4	0.00	1737.8					
*p*_all flowers + richness_	12	1764.1	17.6	0.00	1740.1					
*p*_native + richness_	12	1765.6	19.2	0.00	1741.6					
*p*_distance + native + richness_	13	1772.7	26.2	0.00	1746.7					
*p*_non‐native + richness_	12	1777.9	31.5	0.00	1753.9					
*p*_distance + non‐native + richness_	13	1779.4	33.0	0.00	1753.4					
*p*_richness_	11	1788.2	41.7	0.00	1766.2					
*p*_distance + richness_	12	1796.3	49.8	0.00	1772.3					
*p*_distance + native + non‐native_	13	1813.6	67.1	0.00	1787.6					
*p*_native + non‐native_	12	1816.3	69.8	0.00	1792.3					
*p*_distance + native_	12	1859.9	113.4	0.00	1835.9					
*p*_native_	11	1860.2	113.7	0.00	1838.2					
*p*_distance + all flowers_	12	1861.7	115.2	0.00	1837.7					
*p*_all flowers_	11	1862.6	116.1	0.00	1840.6					
*p*_distance + non‐native_	12	1901.7	155.2	0.00	1877.7					
*p*_non‐native_	11	1902.2	155.8	0.00	1880.2					
*p*_distance_	11	1930.6	184.2	0.00	1908.6					
*p*_(.)_	10	1931.1	184.6	0.00	1911.1					

Note

*K* is the number of model parameters, ΔAIC is difference from top model, w is model weight, and −2LL is the −2*log‐likelihood. Estimated effect sizes (beta estimates ± 1SE) are listed for models with ΔAIC < 10. All models include the general structure, ψ(non‐native + native + richness + distance), *p*(temp2 + humidity + wind + survey‐specific resource variables listed in the model name below).

Using the best‐supported detection (i.e., frequency of use) structure, we investigated factors associated with wild bee use of transects during the growing season (Table 8). The best‐supported model suggested that transects with higher flower richness were more likely to be used by wild bees across the growing season (${\hat{\beta }}_{\mathrm{richness}}$ = 2.3 ± 0.77). The top model also suggested that transects further from apiaries $({\hat{\beta }}_{\mathrm{distance}}$ = 1.4 ± 0.81) and with fewer non‐native flowers were more likely to be used by wild bees $({\hat{\beta }}_{non‐native}$ = −1.4 ± 0.76); however, parameter estimates associated with these covariates were imprecise. The top three supported models all suggested a positive relationship between wild bee use and flower richness (Table 8). Similarly, the top three models showed transects further from apiaries were more likely to be used by wild bees but in all cases parameter estimates associated with the *Distance* covariate were imprecise. Aside from the positive relationship between wild bee use and flower richness, imprecise parameter estimates and model selection uncertainty limited our ability to draw definitive conclusions about wild bee use across the growing season.

**TABLE 8:** Akaike's information criterion (AIC) ranking of candidate models investigating the effect of season‐long flower abundance and richness variables on wild bee occupancy (i.e., probability of use) along 1030, 2 × 20 m transects in North Dakota, South Dakota, and Minnesota, USA, in 2016 to 2017

Model	*K*	AIC	ΔAIC	*w*	−2LL	Ψ_all flowers_	Ψ_native_	Ψ_non‐native_	Ψ_richness_	Ψ_distance_
Ψ_distance + non‐native + richness_	12	1744.4	0.0	0.31	1720.4			−1.4 ± 0.76	2.3 ± 0.77	1.4 ± 0.81
Ψ_distance + richness_	11	1745.9	1.4	0.15	1723.9				1.5 ± 0.84	1.55 ± 0.84
Ψ_distance + native + richness_	12	1746.0	1.6	0.14	1722.0		35.5 ± 37.6		1.5 ± 0.64	1.71 ± 0.90
Ψ_non‐native + native + richness_	12	1746.7	2.3	0.10	1722.7		10.8 ± 4.9	28.0 ± 14.3	−0.20 ± 0.09	
Ψ_all flowers + richness_	11	1746.9	2.4	0.09	1724.9	37.1 ± 65.9			−0.37 ± 0.19	
Ψ_all flowers + distance + richness_	12	1747.3	2.9	0.07	1723.3	89.6 ± 51.4			−0.92 ± 0.69	−0.66 ± 0.97
Ψ_non‐native + richness_	11	1748.1	3.6	0.05	1726.1			−1.3 ± 1.1	1.7 ± 0.94	
Ψ_richness_	10	1748.5	4.1	0.04	1728.5				1.3 ± 0.62	
Ψ_native + richness_	11	1749.8	5.4	0.02	1727.8		18.1 ± 23.3		0.95 ± 0.73	
Ψ_distance + native + non‐native + richness_	13	1751.7	7.2	0.01	1725.7		0.67 ± 2.1	5.7 ± 2.9	−0.25 ± 0.17	5.0 ± 2.5
Ψ_(.)_	9	1754.2	9.8	0.00	1736.2					
Ψ_all flowers_	10	1754.6	10.2	0.00	1734.6					
Ψ_non‐native_	10	1754.7	10.2	0.00	1734.7					
Ψ_native_	10	1755.8	11.4	0.00	1735.8					
Ψ_distance_	10	1756.0	11.6	0.00	1736.0					
Ψ_native + non‐native_	11	1756.5	12.0	0.00	1734.5					
Ψ_distance + all flowers_	11	1756.5	12.1	0.00	1734.5					
Ψ_distance + non‐native_	11	1756.6	12.2	0.00	1734.6					
Ψ_distance + native_	11	1757.7	13.3	0.00	1735.7					
Ψ_distance + native + non‐native + richness_	12	1758.4	14.0	0.00	1734.4					

Note

*K* is the number of model parameters, ΔAIC is difference from top model, w is model weight, and −2LL is the −2*log‐likelihood. Individual beta estimates (±1SE) are listed for models with ΔAIC < 10. All models include the best‐supported detection structure: *p*(non‐native*_j_* + native*_j_* + richness*_j_* + humidity*_j_* + wind*_j_* + temp2).

Collectively, our results indicate that wild bees and honey bees will often occupy the same resource patch, but honey bees more frequently use patches with abundant non‐native flowers, while wild bees more frequently use patches with native flowers and higher flower richness. Our flower visitation data further support this finding, with over 75% of all honey bee observations made on just five forb species: *M*. *officinalis*, *M*. *sativa*, *M*. *alba*, *P*. *tanacetifolia*, and *D*. *purpurea*. These species represented only 19% of wild bee observations.

## 4 DISCUSSION

In this study, we investigated patch use, frequency of use, and flower visitations of wild bees and honey bees in an agricultural landscape that supports the highest density of honey bee colonies in the United States. We found that honey bees were nearly ubiquitous across our study area (grasslands within 7.5 km of known apiaries), suggesting wild bees and honey bees routinely co‐occur among resource patches. Wild bee use of transects across a growing season was most closely related to flower richness. The frequency by which wild bees visited our transects was also positively related to flower richness and abundance. Native flowers increased frequency of use by wild bees to a greater degree than non‐native flowers, a finding supported by other research, demonstrating the importance of native flower diversity and abundance for supporting wild bees (Burkle et al., 2013; McCune et al., 2020; Potts et al., 2003). It is important to note, however, that non‐native flower abundance was also positively related to wild bee frequency of use and >40% of the wild bee observations were made on non‐native flowers. Research from California and New Jersey has shown wild bees will readily use, but not necessarily prefer, non‐native flowers (Williams et al., 2011). Wild bees in the PPR will visit non‐native flowers and even exhibit preference for some, but a majority of the preferred flowers are native (Simanonok et al., 2021). In some cases, non‐native flowers can play a centralized role in maintaining wild bee networks (Larson et al., 2014; Wood et al., 2018) and are often the only plants that grow in highly disturbed soils, typical of agricultural areas (Davis et al., 2000).

Our models revealed that transect frequency of use by honey bees was negatively related to distance to nearest apiary, suggesting that transects closer to apiaries were more frequently visited by honey bees. We found some evidence that wild bees were less likely to use transects that were closer to apiaries; however, the parameter estimates associated with our *Distance* covariate were always imprecise (Table 8), thereby limiting our conclusions. In Europe, researchers have found reduced occurrence and foraging success of wild bees that forage in proximity to commercial apiaries (Henry & Rodet, 2018; Hudewenz & Klein, 2013).

Our results indicated that wild bees and honey bees often co‐occur at the same resource patch but also exhibit a degree of separation when visiting specific flower species within the patch. This finding is supported by the detection (i.e., frequency of use) component of our occupancy analysis, showing wild bee and honey bee detections were more strongly related to native and non‐native flowers, respectively. It is unclear whether differences in floral resource use between honey bees and wild bees observed in our study are due to competitive exclusion, or to differences in resource utilization (Leonhardt & Blüthgen, 2012); however, it seems reasonable to expect wild bees would exhibit preference for native forbs (Morandin & Kremen, 2013), while honey bees would focus foraging efforts on any highly abundant forb. Indeed, *M*.* officinalis*, *M*.* sativa*, and *M*.* alba*, the three most‐visit flower species by honey bees, represent the most abundant forbs in our region (Smart et al., 2021). Honey bees often favor non‐native flowers to those of native flowers but will exhibit preference of some native species (Carr‐Markell et al., 2020). Based on our data, it seems that greatest potential for exploitative competition between honey bees and wild bees in the PPR is with non‐native plants, as only 15% of all honey bee observations were on native flowers. We note our results are specific to the PPR, and region‐specific data should be collected in other portions of the United States where honey bee competition is of concern.

Researchers interested in employing occupancy models to address wild bee resource use should give careful consideration when defining the spatial scale of “resource patches” and the temporal scale at which these resources might change. While our study defined a patch as a single 2 × 20 m transect, future studies may wish to identify resource use at even finer scales such as a single flower, group of flowers, or smaller patch with varying types of resources. Alternatively, for rare species, managers may want to investigate patch use at much larger scales such as entire fields or even entire townships. For example, the US Fish & Wildlife Service is initiating a monitoring program to estimate temporal trends in occupancy of the endangered *Bombus affinis*, where “patches” are a mosaic of 100‐km2 grid cells that span the historic distribution of the species (U.S. Fish & Wildlife Service, 2021). Occupancy models provide researchers and managers with a flexible framework for investigating patch utilization a variety of spatial and temporal scales.

In addition to properly defining the resource patch, the timespan by which repeated surveys are also conducted is important for occupancy studies (MacKenzie et al., 2017). In our research, the availability of floral resources varied considerably during the summer with different species of flowers blooming and senescing at times throughout the growing season. Future studies can improve upon our design by defining periods that better align with the seasonal availability of focal native and non‐native flowering species with replicated surveys within these periods. Incorporating this sampling framework into our study would have provided better understanding of the temporal resource and distributional dynamics of managed and wild bees in our region, and we recommend this consideration for future occupancy studies. Finally, future studies of bee resource use will have to consider whether species or taxon‐specific inferences are desired. Except for a few taxa such as bumblebees, identifying wild bees to species without capturing them is impossible given their small size and similar morphological features. While laboratory identification of wild bees will continue to be required, uncertainty in species identification can also be accounted for within an occupancy modeling framework (Chambert et al., 2015; Miller et al., 2011). Occupancy models that account for species misidentification require that individuals be captured and accurately identified at a subset of patches and surveys, while forgoing capture and unambiguous identification at other locations. This sampling framework has the added benefit of minimizing insect collection and processing effort, a common logistic concern of most wild bee studies (Portman et al., 2020).

Our research suggests opportunities for improving forage for wild bees, while reducing resource overlap with honey bees. The frequency by which honey bees used resource patches was more strongly influenced by non‐native flowers which were three times more abundant at our study sites. This observation is consistent with known honey bee foraging behavior, where colonies will often divert a considerable number of workers to forage on a highly abundant forb (Biesmeijer & Seeley, 2005). In addition to direction‐ and distance‐encoded information in the honey bee waggle dance, individual foraging honey bees can vary the intensity of their dancing to denote attractiveness of floral resources, thus further facilitating the exploitation of abundant flowers (Biesmeijer & Seeley, 2005). Honey bees in particular maintain high flower fidelity during foraging events—often foraging on a single forb species (Amaya‐Márquez, 2009). Land managers seeking to create wild bee habitat without attracting many honey bees may consider keeping the diversity of forbs high and the density of any individual species low to moderate so that no forb species becomes highly abundant once the planting is established. The total number of seeded forbs can be moderate to high to ensure proper establishment, but species evenness should also be maximized such that no single forb species becomes dominant and therefore a forage target of honey bees. Our research also suggests areas with native forbs will see more routine use by wild bees, relative to areas with non‐native forbs. Based on our data, it appears maximizing benefits to wild bees, while reducing competitive interactions with honey bees, is most likely to be achieved through seeding native forbs, with proper management to ensure no single forb species dominates the field.

Alternatively, new pollinator habitat can be partitioned such that a single field includes multiple plots, each containing a separate seed mix: one to attract honey bees and the other to attract wild bees. Partitioning habitat for honey bee and wild bees has recently been adopted by pollinator habitat organizations such as the Bee and Butterfly Habitat Fund (https://beeandbutterflyfund.org/), which offers multiple seed mixes to private landowners to provide native flower patches for native bees and butterflies, and legume‐rich, non‐native flowers as honey bee forage. These separate seeding mixes are then applied to different fields within a farmstead. Although empirically untested, the idea of subdividing fields into multiple seed mixes to reduce resource overlap between honey bees and wild bees provides a potentially attractive option to managers. Improving pollinator forage in agroecosystems of the PPR has the added benefit of alleviating potential conflicts between commercial beekeepers and conservation groups elsewhere in the United States because beekeepers will not be pressured to seek high‐quality forage sites in more unaltered, public lands (Wojcik et al., 2018).

## CONFLICT OF INTEREST

The authors have no competing interests to declare.

## AUTHOR CONTRIBUTIONS

**Clint R. V. Otto:** Conceptualization (lead); Data curation (lead); Formal analysis (lead); Funding acquisition (lead); Investigation (lead); Methodology (equal); Project administration (lead); Resources (lead); Software (lead); Supervision (lead); Validation (lead); Visualization (lead); Writing‐original draft (lead); Writing‐review & editing (equal). **Larissa L. Bailey:** Formal analysis (supporting); Software (supporting); Validation (supporting); Writing‐review & editing (equal). **Autumn Smart:** Conceptualization (supporting); Data curation (supporting); Investigation (supporting); Methodology (supporting); Writing‐review & editing (supporting).

## Figures and Tables

**FIGURE A1:**
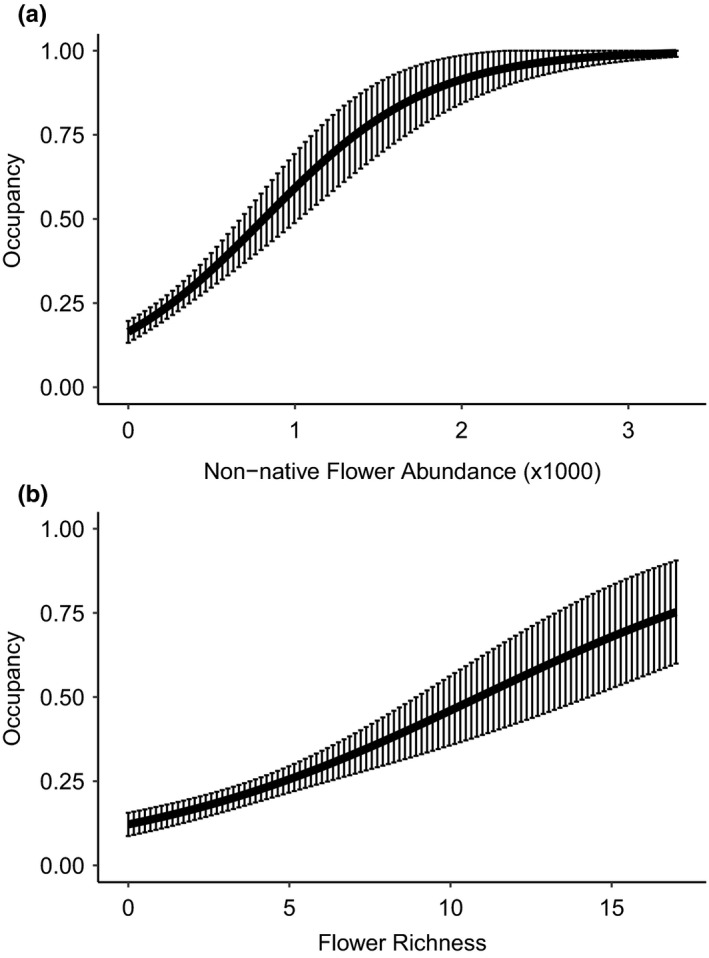
Honey bee frequency of use (detection probability) modeled as a function of the total number of (a) non‐native flowers and (b) flower richness during a survey along 2 × 20 m grassland transects in North Dakota, South Dakota, and Minnesota, USA, in 2016 and 2017. Estimates were based on the best‐supported model Ψ (non‐native + native + richness), *p*(distance + non‐native*_j_* + native*_j_* + richness*_j_* temp2*_j_* + cloud*_j_* + humidity*_j_*), using the mean value for the other covariates. Covariate axes were truncated to show most of our data points (Table 1). Error bars are ± 1SE

**FIGURE A2:**
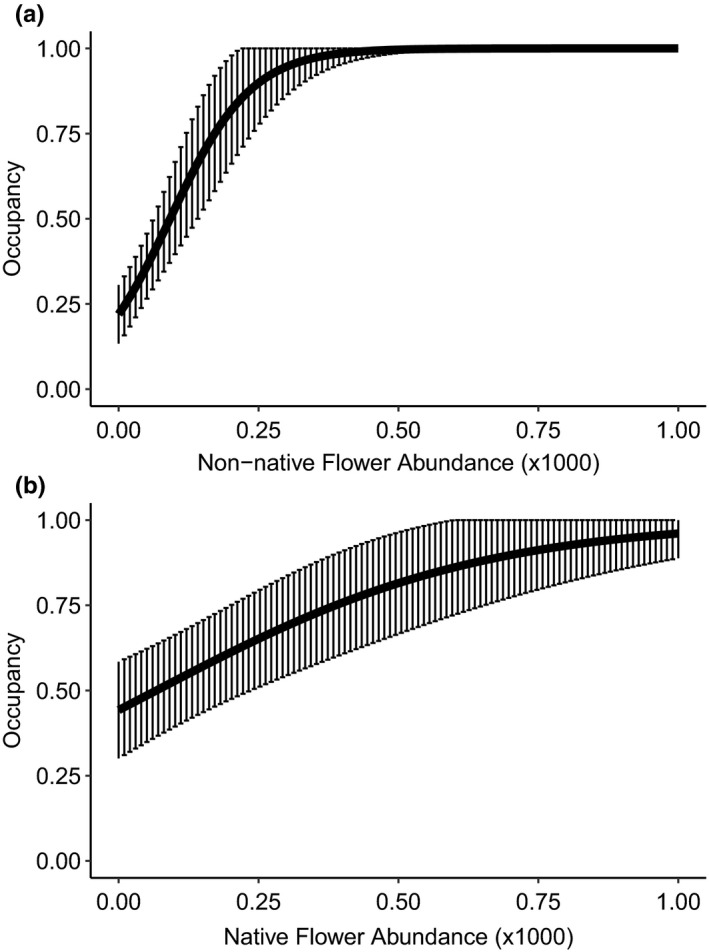
Honey bee occupancy estimates (i.e., probability of use) as a function of the abundance of A) non‐native and B) native flowers counted along 2 × 20 m grassland transects in North Dakota, South Dakota, and Minnesota, USA, in 2016 and 2017. Estimates were based on the best‐supported model Ψ (on‐native + Native), *p*(distance + non‐native*_j_* + native*_j_* + richness*_j_* temp2*_j_* + cloud*_j_* + humidity*_j_*), using the mean value for the other covariates. Covariate axes were truncated to show most of our data points (Table 1). Error bars are ± 1SE

**FIGURE A3:**
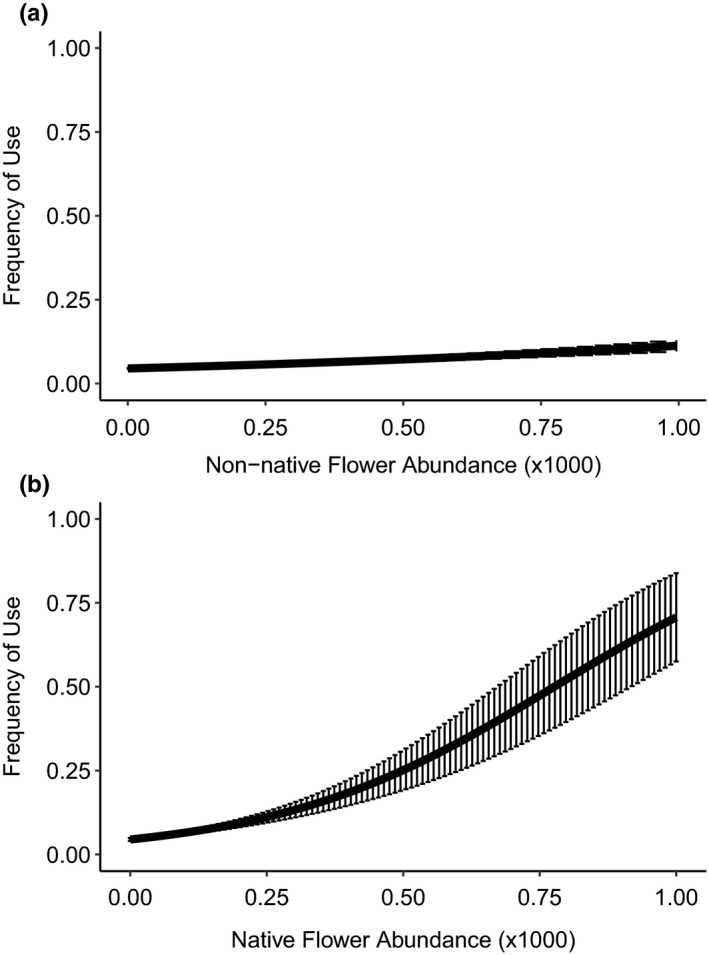
Wild bee frequency of use (detection probability) explained as a function of the number of (a) native and (b) non‐native flowers counted during a single survey along 2 × 20 m grassland transects in North Dakota, South Dakota, and Minnesota, USA, in 2016 and 2017. Estimates were based on the best‐supported model Ψ (non‐native + native + richness), *p*(non‐native*_j_* + native*_j_* + richness*_j_* + temperature2 + wind), using the mean value for the other covariates. Covariate axes were truncated to show most of our data points (Table 1). Error bars are ± 1SE

## Data Availability

All data and metadata associated with this project are available in a USGS data release: https://doi.org/10.5066/P9O61BCB.
